# Influence of Microbiota on Intestinal Immune System in Ulcerative Colitis and Its Intervention

**DOI:** 10.3389/fimmu.2017.01674

**Published:** 2017-11-28

**Authors:** Sai-Long Zhang, Shu-Na Wang, Chao-Yu Miao

**Affiliations:** ^1^Department of Pharmacology, Second Military Medical University, Shanghai, China

**Keywords:** intestinal microbiota, host immune response, ulcerative colitis, epithelial cells, therapeutic targets

## Abstract

Ulcerative colitis (UC) is an inflammatory bowel disease (IBD) with chronic and recurrent characteristics caused by multiple reasons. Although the pathogenic factors have not been clarified yet, recent studies have demonstrated that intestinal microbiota plays a major role in UC, especially in the immune system. This review focuses on the description of several major microbiota communities that affect UC and their interactions with the host. In this review, eight kinds of microbiota that are highly related to IBD, including *Faecalibacterium prausnitzii, Clostridium* clusters IV and XIVa, *Bacteroides, Roseburia* species, *Eubacterium rectale, Escherichia coli, Fusobacterium*, and *Candida albicans* are demonstrated on the changes in amount and roles in the onset and progression of IBD. In addition, potential therapeutic targets for UC involved in the regulation of microbiota, including NLRPs, vitamin D receptor as well as secreted proteins, are discussed in this review.

## Introduction

Ulcerative colitis (UC) is a sort of chronic recurrent disorder with the characteristics of intestinal mucosa inflammation and ulceration (1–3). The disease causes significant morbidity worldwide, with morbidity and prevalence increasing over time (4). UC is also regarded as a polygenic and multiple diseases caused by a series of complex factors such as environment, genes, and immunomodulatory factors (5).

The microenvironment of the gut forms a good microbiota habitat, which has been demonstrated to affect many physiological conditions in earlier studies (6–8). Since intestinal microbiota is considered as an important organ of the human body in recent times, an increasing number of studies have linked this microenvironment to gastrointestinal diseases. Because the composition of the intestinal microbiota is stable over a period of time, many studies inferred the gut microbiota as a potential predictor of health status and a target for therapeutic intervention (9). Moreover, it has been report that intestinal microbiota has a key role in inflammatory bowel disease (IBD), including UC and Crohn’s disease (CD) (10). Considering the complexity and diversity of the human gut microbiota, there is no denying that it is difficult to demonstrate the presence of specific bacterial strains, which play a certain role in the pathogenic mechanism of IBD. Unlike the well-researched CD, our knowledge on UC is relatively deficit, and there remain many contradictions to be elucidated.

In this review, the relationship between UC and several popular microbiota communities highly related as well as the potential therapeutic targets for UC involved in the regulation of microbiota will be discussed.

## Part I: Microbiota

**Figure 1:**
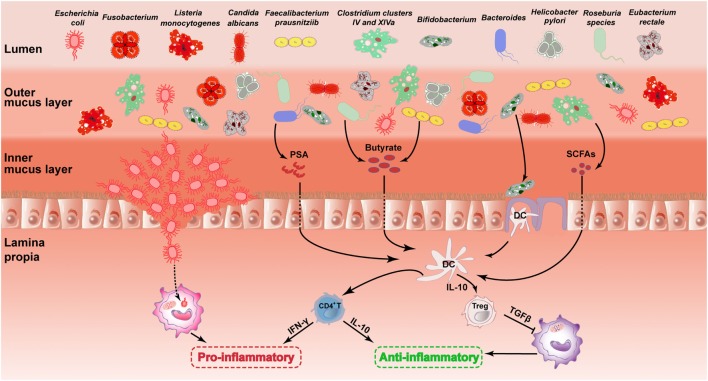
Role of the gut microbiota in the pathogenesis of UC. The picture describes the changes in the major intestinal microbiota in the UC and its influence on gastrointestinal. This illustration contains 11 types of intestinal microbiota and their changes mentioned in this review. The mechanisms underlying the effects of certain microbiota on the gastrointestinal are described. It includes microbiota acting on DC cells by secreting substances such as PSA, butyrate and SCFAs. Then, DC cells further act on CD4^+^ T cells or regulatory T (Treg) cells to inhibit inflammation. There are also mechanisms by which AIEC destroys the gut barrier and further induces inflammation. The first four species of microbiota are painted in dark red, representing harmful microbiota. The other seven species of microbiota are painted in other colors, representing healthy microbiota. UC, ulcerative colitis; DC, dendritic cell; PSA, polysaccharide A; SCFA, short-chain fatty acid; AIEC, adherent-invasive *Escherichia coli*.

The colon has two mucus layers, which is different from the small intestine with a single layer of mucus. The inner layer is a mucous lining that is closely linked to the intestinal epithelium, which provides a sterile environment. Outer layer is a mucous layer of varying thickness, composed of mucins, trefoil peptides, and secretory IgA (11, 12). Although there is bidirectional effects between the microbiota and the host, its direct effects on intestinal epithelial cells are limited by mucus layers and antimicrobial peptides (AMPs) such as defensins and regenerating islet-derived 3 gamma (Reg3g) (7, 13, 14). The healthy and complete mucus layer only enables intestinal microbiota to attach to the mucus layer instead of the direct touch of intestinal epithelial cells. There are four phyla of microbiota in normal human intestine including *Bacteroidetes, Firmicutes, Actinobacteria*, and *Proteobacteria*, two of which (*Bacteroidetes* and *Firmicutes*) are dominant in the gut (15–17). In the intestinal tract of healthy people, *Firmicutes*, a community of Gram-positive bacteria, are classified into two main groups: *Bacilli* and *Clostridia* (primarily *Clostridium* cluster IV and *Clostridium* XIVa). The Gram-negative *Bacteroidetes* resides in the gut as one of the most abundant genera. And some studies have shown that these four groups are relatively stable in healthy people (9, 18). Here, in this section, we focus on the description of the microbiota which are closely related to the pathogenesis and progression of UC (summarized in Table 1 and Figure 1).

**Table 1:** Changes of potential beneficial and harmful microbiota in UC.

	Changes in UC	Mechanisms	Reference
**Beneficial microbiota**			
*Faecalibacterium prausnitzii*	↓	Enhancing production of Treg cells, energy supply of intestinal epithelial cells and IL-10	(30–35, 38, 40–44)
*Clostridium* clusters IV and XIVa	↓	Producing butyrate	(34, 43–45, 58)
*Bifidobacterium*	↓	Inhibiting intestinal inflammation by acting on Treg cells	(101, 102)
*Bacteroides*	↓	Inducing CD4^+^ cells by enhancing IL-10 and IL-17 through secreting PSA	(69, 70)
	↑	Invade intestinal tissues and cause damage	(67)
*Helicobacter pylori*	↓	5-ASA and antibiotics	(105–108)
	↑	Epidemiological data showed no significant correlation	(109, 110)
*Roseburia* species	↓	Producing butyrate	(44, 46)
*Eubacterium rectale*	↓	Unknown	(33, 44)
**Harmful microbiota**			
*Escherichia coli* (adherent invasive)	↑	Invading intestinal epithelial cells, replicating in macrophages and inducing granulomas	(21, 94–97)
	—	Inactive UC patients	(87, 88, 93)
*Fusobacterium*	↑	Unknown	(38, 112–116)
*Listeria monocytogenes*	?	Unknown	(117, 118)
*Candida albicans*	↑	Unknown	(119, 120)

*PSA, polysaccharide A; UC, ulcerative colitis; Treg, regulatory T*.

*↓, Decrease; ↑, increase; —, unchange; ?, to be determined*.

### *Faecalibacterium* *prausnitzii*

The *F. prausnitzii* is one of the richest species. There are large population of *F. prausnitzii* bacteria in the normal human body, occupying 6–8%, even 20% among all kinds of microbiota (17, 19–23). Some studies reported it as one of the main producers of a short-chain fatty acid (SCFA) called butyrate in the intestine. SCFA has an anti-inflammatory effect that results from the production of regulatory T (Treg) cells and the energy supply of intestinal epithelial cells (24, 25). In addition, it (SCFA) also exerts anti-inflammatory effects through upregulating the anti-inflammatory cytokines secretion such as IL-10 (26, 27). It was further reported that the anti-inflammatory effects are partially related to their disruption of NF-κB activation, blocking IL-8 production (28, 29). Based on those previous studies, *F. prausnitzii* plays an important role in the protection of colonic functions through its anti-inflammatory mechanisms.

In recent studies, *F. prausnitzii* has been demonstrated to involve in the maintenance of intestinal health (26, 29–46). Of note, some studies have shown that there is a significant difference in *F. prausnitzii* between healthy people and UC patients (30, 31, 33–38, 41, 46). Compared with healthy people, *F. prausnitzii* species had lower counts in UC patients (30–35, 38, 40–44). For instance, Machiels et al. introduced real-time PCR analysis to find that *F. prausnitzii* in UC patients had a lower abundance than health people. They also demonstrated an inverse correlation with disease activity (46). Furthermore, Varela et al. found that less than 12 months of remission and more than one relapse/year were linked with low counts of *F. prausnitzii*. And the recovery of the *F. prausnitzii* population after relapse has connection with clinical remission maintenance (42). In fact, phylogenetic analysis showed that there were at least two subtypes of *F. prausnitzii*, with differences in the distribution of subtypes among people with gut disorders and healthy subjects (32, 39). Phylogroup I was accounted for 87% in healthy subjects while under 50% in IBD patients. By contrast, phylogroup II was found in IBD patients with >75%, while only 52% in healthy subjects. This study reveals that even though the majority of the *F. prausnitzii* population exists in both healthy subjects and gut diseases individuals, the latter has a reduced richness and an altered phylotype distribution exists among diseases (39). So far, it is unknown whether there are any other subtypes within the species or other *Faecalibacterium* species. Besides the researches in patients, there are also some studies in experimental data. For instance, Martín et al. reported that administration of *F. prausnitzii* led to a significant decrease in IBD severity in both severe and moderate chronic colitis models. They also demonstrated that this kind of microbiota could prevent physiological damage in a chronic low-grade inflammation murine model (47, 48). Furthermore, Zhang et al. found that *F. prausnitzii* inhibits interleukin-17 to ameliorate colorectal colitis in rat (49).

Although some studies have shown the characteristics of *F. prausnitzii*, the influential factors of *F. prausnitzii* still remain to be unclarified. For instance, treatment with mesalazine and immunosuppressive agents did not restore the number of *F. prausnitzii* (37). On the contrary, it was also reported that the plenty of *F. prausnitzii* was increased in time of induction therapy in patients (36).

### *Clostridium* 

*Clostridium* is a community of Gram-positive bacteria, which includes several significant human pathogens such as the causative agent of botulism and an important cause of diarrhea, *Clostridium difficile*. *Clostridium* species normally inhabit in animal and human soil and intestinal tract. It also exists as a normal resident in the healthy lower reproductive tract of women (50). There are three major species (*C. difficile, Clostridium coccoides*, and *Clostridium leptum*) of *Clostridium* species related to UC.

As a Gram-positive bacterium, *C. difficile* could produce toxins and cause colitis, especially in patients with antibiotic treatment, resulting in the destruction of commensal microbiota (51, 52). Toxins secreted by *C. difficile* can lead to a severe effect on intestinal mucosa. Of note, it has been reported that *Clostridium difficile* infection (CDI) led to the damage of intestinal barrier through the secretion of exotoxin (53). CDI has risen sharply in the past two decades. IBD patients are more likely to be infected with *C. difficile* because of immunosuppression and dysbiosis *in vivo* (54–56). Singh et al. showed that individuals with IBD had a 4.8-fold increase in risk of CDI than individuals without IBD and there is no difference between individuals with UC vs. CD (55). Interestingly, Mabardy et al. reported a decrease of mortality for IBD and non-IBD patients with *C. difficile* but a greater decrease in mortality for IBD patients (54). The incidence and severity of IBD associated with CDI are significant, especially in UC patients with colonic involvement, where the probability of surgery is 20% (57). Notably, *C. difficile* has been discussed to cause the relapse of IBD (58). The main problem raised by the current study is that CDI is produced after antibiotic treatment (59, 60). UC patients may have a higher risk of infection with *C. difficile* because of antibiotics and immunomodulatory drugs. However, it is worth mentioning that, in addition to the potential pathogenicity of *C. difficile*, there is no direct evidence that *C. difficile* can cause UC.

On the contrary, the other two *Clostridium* species are *C. coccoides* (also called *Clostridium* clusters XIVa) and *C. leptum* (namely, *Clostridium* clusters IV), which appears to be credible factor related to UC (34, 43, 44). *Clostridium* clusters IV and XIVa were demonstrated to play a significant role in maintaining intestinal function by producing butyrate (45, 61). Furthermore, it was reported that those two microbiota were reduced in the occurrence of UC, suggesting a potential therapeutic target in the treatment of UC (43, 44). For instance, *C. leptum* was reported to induce murine tolerogenic dendritic cells (DCs) and Treg cells *in vitro* while disrupting the immune inflammatory response (62).

### *Bacteroides* 

*Bacteroides* are a genus of Gram-negative, obligate anaerobic bacteria, which are important components of the mammalian gut commensal bacteria. *Bacteroides* species are non-endospore-forming bacilli and may be either motile or non-motile depending on the species of the host. *Bacteroides* species are usually genetically diverse and constitute the dominant microbiota in the mammalian gastrointestinal tract, playing a fundamental role in processing complex molecules in the host gut to simple molecules (63, 64).

The bacteria can exert its beneficial effects on the host by immune regulation and maintenance of homeostasis. It was reported that UC showed an increase of the number of *Bacteroides fragilis*, which could enhance the mRNA expression of anti-inflammation-related cytokines such as IL-10, through the secretion of polysaccharide A (PSA) (65–68). Colonized *B. fragilis* can reverse CD4^+^ T-cell defects and Th1/Th2 imbalance in germ-free mice (65). Furthermore, it was suggest that *B. fragilis* could protect from experimental colitis, possibly by inducing CD4^+^ cells *via* IL-10 (69, 70). For instance, Round et al. reported that *B. fragilis* directed Foxp3^+^ Tregs development and germ-free animal monocolonization by augmenting the suppressive capacity of Tregs and inducing the anti-inflammatory cytokines production solely from Foxp3^+^ T cells in the gut (70). Besides those researches conducted on patients, experimental data were obtained in animal models. For instance, there has been reported that *Il10^−/−^* mice had increased proportions of *Bacteroides* species (71). In addition, Okayasu et al. found that the amount of *Bacteroides* was significantly increased in mice with acute and chronic UC (72).

Based on the above evidence, we believe that *B. fragilis* is critical for maintaining a healthy physiological state of the host. However, *B. fragilis* is not entirely beneficial in UC. Contrary to previous reports, it was reported to invade intestinal tissues and cause damage in individual patients (73). In addition, other genera of *Bacteroides* (*Bacteroides vulgatus* and *Bacteroides ovatus*) were also found to influence IBD progression (74–80).

### *Escherichia* *coli*

*Escherichia coli*, usually found in the lower intestine of warm-blooded organisms, belongs to the family of *Enterobacteriaceae* (a large family of Gram-negative bacteria that includes many harmful species). As a commensal bacterium widely found in vertebrates, it infected many people each year through intraintestinal and extraintestinal pathways and was reported to kill more than two million humans per year (81). Because *E. coli* can be transmitted in water and sediment, it is often used as a test indicator of water pollution. The temperature and nutrients in these environments can support the viability of saprophytic *E. coli* (82, 83).

It has been reported that the number of *E. coli* is elevated in UC, whether in mouse models or UC patients (21, 84–89). For instance, the number of *E. coli* at the inflammatory sites of UC patients showed a significant increase compared with the control group. Meanwhile, comparative analysis method was introduced to analysis the restriction patterns of *E. coli* isolated from inflammatory and unchanged tissues, respectively. Those results showed that the local inflammatory change did not promote specific strains of *E. coli* (87). Siczek et al. also demonstrated that administration of NanoAg2 could reduce the number of *E. coli* alleviating colitis in experimental models of UC (90). Some other reports, however, showed that the number of *E. coli* did not rise significantly compared with healthy controls (41, 91, 92). Considering the increased number of *E. coli* has connection with the activity status of UC patients, the difference in the severity of UC may account for the reason. For instance, more abundant of *E. coli* were found in active UC patients than in inactive UC patients or healthy people (87, 88, 93).

*Escherichia coli* from ileal CD patients, particularly adherent-invasive *Escherichia coli* (AIEC) strains, have been reported to be enriched in UC patients (94, 95). The concentration of *E. coli* in mucosal sample is larger than that in fecal sample (96). The invasive ability of AIEC strains allows bacteria to move through the human intestinal barrier to the deep tissues. Furthermore, AIEC is able to invade intestinal epithelial cells, replicate in macrophages and induce granulomas *in vitro* (97). AIEC adheres to an N-glycosylated chitinase 3-like-1 on IECs *via* the chitin-binding domain of chiA promoting the pathogenic effects of AIEC in mice with colitis (98). The number of AIEC can affect the course of the disease. In addition, mesalazine, an anti-inflammatory drug, can decrease the number of AIEC and relieve inflammation in patients with IBD (99). Therefore, mesalazine might serve as a potential therapeutic strategy in the treatment of UC caused by AIEC infection.

### Others

*Bifidobacterium*, a community of anaerobic Gram-positive genus, is everywhere in the intestine (100). *Bifidobacterium* is widely used as one of probiotics, and studies have demonstrated that it plays a significant role in the UC treatment. It was demonstrated that oral administration of *Bifidobacterium* could block intestinal inflammation by acting on Tr1 cells, leading to the production of IL-10, thereby ameliorating colitis in immunocompromised mice (101, 102). However, so far, since we have little knowledge on the relationship between *Bifidobacterium* and UC, further studies are demanded on this issue.

*Helicobacter pylori* is found in the gastrointestinal tract, characterized by its microaerophilic metabolism and spiral shape (103, 104). Although *H. pylori* is widespread in the epithelium of the stomach, studies have shown that it can also be detected in the colon and fecal in UC patients (105–108). The relationship between UC and *H. pylori* is contradictory and complex. For example, it was demonstrated by Engler et al. (109) that infection or treatment with *H. pylori* significantly alleviated the severity of DSS-induced chronic colitis as well as T-cell transfer-induced colitis both in clinical symptom and histopathological features, indicating the protective role of *H. pylori* in UC. However, some epidemiological data showed that there was no significant correlation between *H. pylori* and UC (110, 111). Thus, future studies are needed to distinguish between a true protective role of *H. pylori* and the outcome of UC.

*Fusobacterium* species are anaerobic Gram-negative bacteria, which are members of the normal microbiota of the oral and intestinal tract. However, certain species of *Fusobacterium*, such as adherent, invasive, pro-inflammatory species, were identified as UC pathogens (38, 112, 113). The relative abundance of *Fusobacterium* in the colonic mucosa of mouse colitis models or IBD patients is increased, among which 69% of all IBD patients-derived *Fusobacterium* were identified as *Fusobacterium nucleatum* (114–116). Moreover, commensal bacteria, including *Fusobacterium varium* that was one of mucosal organisms isolated from UC patients, were found as a possible pathogen in UC (112, 113).

Apart from those above microbiota, there are some other species relate to the pathogenesis and progression of UC, including *Roseburia hominis, Eubacterium rectale, Listeria monocytogenes*, and *Candida albicans* (33, 35, 44, 46, 117–120). For instance, in UC patients, *R. hominis*, a well-known kind of *Firmicutes* phylum bacteria that produces butyrate, differs from that of healthy individuals in number (44, 46). *E. rectale* was reported to be significantly reduced in abundance (33, 44), especially in pediatric UC (35). The Gram-positive *L. monocytogenes* is an intracellular pathogen and is often transmitted to other sites. In recent years, studies have found that it may exacerbate the severity of UC (117). However, in UC endoscopic biopsy samples, it was shown that the number of *L. monocytogenes* did not differ from the normal group. As a result, it does not stand by the direct action for *L. monocytogenes* in the IBD pathogenesis (118). *C. albicans* is most often isolated in 91.7% patients in UC and can delay healing of UC (119, 120).

## Part II: Potential Targets for UC Therapy

**Figure 2:**
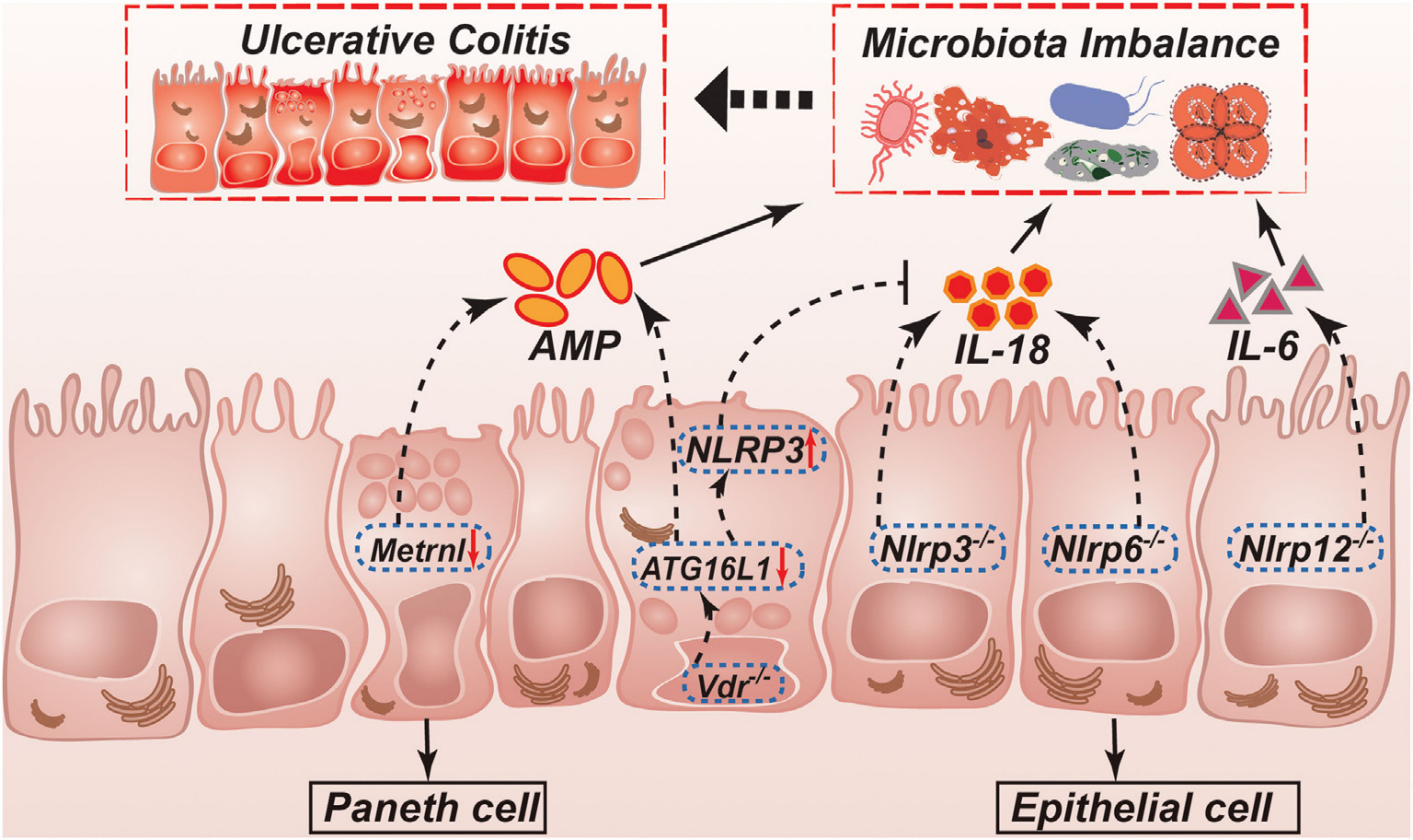
Possible mechanisms controlled by NLRPs, VDR, and Metrnl in regulation of intestinal homeostasis and ulcerative colitis. NLRP3 and NLRP6 inflammasomes regulate secretion of IL-1β and IL-18. IL-18 helps to maintain a non-pathogenic gut microflora, which promote a healthy gut environment. IL-18 is not produced in *Nlrp3*^−/−^ or *Nlrp6*^−/−^ mice, leading to the development of potentially pathogenic species. *Nlrp12*^−/−^ mice results in a more inflammatory environment caused by higher production of cytokines such as IL-1β and IL-6. ATG16L1 is decreased in *Vdr*^−/−^ mice, which leading to reduction of AMP. Furthermore, ATG16L1 decreasing can also inhibit IL-18 production through upregulating NLRP3 expression. In the intestinal epithelial cell-specific Metrnl knockout mice, reduction of AMP leading to microbiota imbalance. NLRP, NLR family, pyrin domain-containing; VDR, vitamin D receptor; AMP, antimicrobial peptide.

In the above contents, the roles and changes of several kinds of microbiota in UC have been fully discussed in above contents. Since intestinal microbiota are important in the onset and development of IBD, searching for new intervention targeting microbiota may have potential therapeutic implications for the treatment of UC. It has been reported that interventions such as NLRPs and vitamin D receptor (VDR) may affect the pathogenesis and progression of UC through regulating the composition of microbiota. In addition, several kinds of secreted proteins have been demonstrated to serve as potential therapeutic targets for UC. In this section, we will focus on recent studies on the targets of microbiota, which might be potential therapeutic strategies in the treatment of UC (illustrated in Figure 2).

### NLRPs

NOD-like receptor (NLR) family is one of the important groups of PRRs in IBD. The number of NLRs variety in human and mice are 22 and 30, respectively (121). They are characterized by central nucleotide binding and oligomerization domain. NLRPs (NLR family, pyrin domain containing) can activate caspase-1 and start the inflammatory process (122).

The NLRP3 inflammasome is the most clarified inflammasome and has been reported to further affect disease progression in UC by influencing microbiota (123). Previous reports showed the changes in the number and composition of microbiota in *Nlrp3*^−/−^ mice. *Nlrp3*^−/−^ mice have been reported to have larger number of microbiota in colon than the wild-type mice (124). Hirota et al. showed that the number of *Firmicutes* and *Proteobacteria* are increased in the colon from *Nlrp3*^−/−^ mice compared with the wild-type group (125). Although *Nlrp3*^−/−^ mice were demonstrated to be highly susceptible to DSS-induced UC in several studies, one study put forward the contrary results proving that deficiency of the NLRP3 inflammasome protected mice against DSS (124–127). Therefore, further efforts are demanded in the determination of the effects and mechanisms of the NLRP3 inflammasome in UC so that we are able to take advantage of the NLRP3 inflammasome in the treatment of UC in the future.

The expression of the NLRP6 inflammasome can be detected in both the large intestine and the small intestine. It was reported that *Nlrp6*^−/−^ mice more susceptible to UC induced by DSS with tissue injury, bleeding, and increased permeability of the gut epithelium (126, 128). It was supposed that the changes in microbiota population of these mice might result from aberrant host–pathogen interactions at this site (129).

The NLRP12 inflammasome has also been found to be participated in the inflammatory process of the colon, especially the protective role in acute colitis. *Nlrp12*^−/−^ mice exhibited more severity in colitis upon DSS administration than wild-type mice (130, 131). For instance, Zaki et al. showed that *Nlrp12*^−/−^ mice were highly vulnerable to colon inflammation, indicating the connection with increased production of inflammatory cytokines including IL-1β and IL-6. Those effects were led to by the activation of inflammation-related pathways such as NF-κB and ERK signals in colonic macrophages.

The current major problem is that we have not yet been able to figure out its downstream mediators of the NLRPs and their inflammasomes. It is not clear which factors mediate the effects and whether the observed results are related to a specific microbiota or a combination thereof.

### Vitamin D Receptor

Vitamin D is a group of lipid-soluble steroid substances that promotes the absorption of calcium and magnesium ions. Besides, it has various biological effects, including depression, cardiovascular disease and cancer (132–134). The VDR as a nuclear receptor of vitamin D plays an important role in regulating intestinal microbiota homeostasis and commensal bacteria living environment (135–139). It has been reported that VDR signaling pathway abnormalities and low expression are related to UC (140–143).

Vitamin D can not only regulate the immune response to intestinal microbiota but also change the composition of microbiota (144, 145). For instance, using genome-wide association analysis, two cohort studies showed that the variation of *Vdr* gene in the host could affect the intestinal microbiota through the measurement of selected bile and fatty acids in humans (146). Chen et al. reported that the levels of *Eubacterium, Bacteroides*, and *Salmonella* were significantly different between *Vdr*^−/−^ and wild-type mice (147). It was also demonstrated in another study that the number of *Firmicutes* was decreased and that of *Bacteroides* and *Proteobacteria* were increased in the fecals in *Vdr*^−/−^ mice (144). Jahani et al. showed that vitamin D deficiency at birth caused lower number of colonic *Bacteroides* and *Prevotella* later in life (148). Moreover, *Vdr*-deficient mice would cause bacterial imbalance, such as depletion of *Lactobacillus*, as well as enrichment of *Clostridium* and *Bacteroides*. In addition, those changes might lead to alterations in some important pathways and further cause other diseases (149). Interestingly, giving vitamin D could lead to an increased number of colon *Citrobacter rodentium* through inhibiting the Th17 response (150). Remarkably, the lack of vitamin D diet could cause intestinal barrier dysfunction, which substantially made the body more susceptible to infection by intestinal microbiota (151). Similarly, immune changes might cause *Vdr*-deficient mice to become more sensitive to non-pathogenic bacteria and increase sensitivity to *Salmonella*-induced colitis inflammation (152, 153). Moreover, lacking *Vdr* will cause dysbiosis and resistance to colonization by *C. rodentium* through strengthening IL-22-producing innate lymphoid cells (154).

So far, although the positive effects of vitamin D system have been proven on the regulation of intestinal microbiota in animal studies, the evidence of those positive effects in human experiment were deficient in the UC patients. Thus, to ultimately take advantage of intestinal microbiota in the treatment of UC, further basic researches and clinical studies should be conducted on this issue.

### Secreted Proteins

Secreted proteins belong to a large family which can be secreted by a cell whether in an endocrine or exocrine manner. Among all of the secreted proteins, cytokines are the most studied proteins. Since first reported in 1965, cytokines were regarded as loose and broad proteins with molecular weight of 5–20 kDa and was demonstrated to play vital roles in the cellular activity (155). As we discussed earlier, several kinds of microbiota could affect intestinal barrier status through regulating the secretion of various kinds of cytokines, thus producing effects on the pathogenesis and progression of UC. For instance, *F. prausnitzii* could upregulate the production and secretion of IL-10 while inhibiting the secretion of IL-8 (26, 27, 29). In addition, *Bacteroides* and *Bifidobacterium* were reported to act on DCs to regulate the levels of IFN-γ and TGF-β secreted from CD4^+^ T cells and Treg cells (156). Of note recent studies have reported that several kinds of cytokines, such as IL-1β and IL-10, have an impact on intestinal microbiota and subsequently led to the regulation of UC (157, 158). It was demonstrated that interferon-β, an NLRP3 inflammasome inhibitor and its secretion of IL-1β and IL-18, contributed to the amelioration of gut inflammation and maintenance of gut homeostasis (159). Taken together, taking advantage of targeting to those cytokines might become a potentially effective therapeutic strategy in the UC treatment.

Beside cytokines, another kind of important secreted proteins regulating the gut homeostasis is adipokine, which are secreted by adipocyte. For example, Metrnl, one of the adipokines, has been reported by studies in our lab which showed a potential regulatory effect on the pathogenesis and development of UC. Metrnl belongs to a secreted protein containing 311 amino acids. It has a similar function to its only homologous gene, Meteorin (Metrn), which is a novel neurotrophic factor (160, 161). Recombinant Metrnl protein can accelerate neuroblast migration *in vitro* and display a neuroprotective effect *in vivo* (162). A previous study in our lab identified Metrnl as a novel adipokine during a process to screen new adipokines, demonstrating that Metrnl was abundant in the subcutaneous adipose tissue in both humans and mice (163, 164). Meanwhile, we found that Metrnl had higher expression in the human gastrointestinal tract, specifically expressed in the intestinal epithelium (165). Consistently, the mRNA of Metrnl in the mouse gastrointestinal tract also had the highest expression among the tested 14 types of tissues. In the intestinal epithelial cell-specific Metrnl knockout mice, the Metrnl level in the gut fluid were significantly reduced, whereas the Metrnl level in the serum showed a decreasing tendency with no statistical significance. The cell-specific Metrnl deletion did not affect physiological conditions of body weight, food intake, blood glucose, colon length and histology, intestinal permeability, mucus content, or mucin 2 expression but significantly decreased AMPs expression, such as Reg3g and lactotransferrin (165). As AMPs are closely related to microbiota balance, Metrnl may become a potential target for UC treatment. However, developing the related treatment is still a long way to go.

## Conclusion and Perspectives

So far, different genera of microbiota have been described. It is widely acknowledged that intestinal microbiota plays a significant role in the pathogenesis and progression of UC. It has been reported that certain single microbiota or a combination of individual microbiota may serve as the one of the causes of UC. Furthermore, more and more studies have shown that there is a high prevalence of certain bacterial species in UC patients. However, our understanding on the pathogenicity of the individual microbiota and the human host is still limited. So far, none of the bacteria have been specifically shown to be the direct cause of UC because the majority of the related studies were conducted only after the onset of disease. As we discussed earlier, several strategic targets for UC involved in microbiota were reported taking advantage of their anti-inflammatory effects. However, the mechanisms underlying the treatment of bacterial populations in specific hosts are not very clear, or that molecular targets are not specific. Studies have shown that restoring balance between the host and microbiota can reduce the incidence of UC. However, it is impossible to completely restore the microbiota, and the removal of certain pathogenic bacteria is not sufficient to achieve a good therapeutic effect. Therefore, it is necessary to further understand the molecular basis of host and bacterial interaction and provide a good strategy for the treatment of microbiota in the future.

## Author Contributions

S-LZ retrieved and analyzed concerned literatures. S-LZ and S-NW wrote the manuscript. C-YM revised the manuscript. All the authors agreed to be accountable for the content of the work.

## Conflict of Interest Statement

The authors declare that there is no conflict of interests regarding the publication of this paper. The reviewer KL and handling editor declared their shared affiliation.
